# Environmentally Friendly Techniques and Their Comparison in the Extraction of Natural Antioxidants from Green Tea, Rosemary, Clove, and Oregano

**DOI:** 10.3390/molecules26071869

**Published:** 2021-03-26

**Authors:** Mariel Calderón-Oliver, Edith Ponce-Alquicira

**Affiliations:** 1 Tecnologico de Monterrey, Escuela de Ingeniería y Ciencias, Avenida Eduardo Monroy Cárdenas 2000, San Antonio Buenavista, Toluca de Lerdo 50110, Mexico; mariel.calderon.oliver@tec.mx; 2 Departamento de Biotecnología, Universidad Autónoma Metropolitana, Unidad Iztapalapa, Avenida San Rafael Atlixco 186, Col. Vincentina, Ciudad de Mexico 09340, Mexico

**Keywords:** polyphenols, extraction, environmentally friendly, antioxidant, oregano, rosemary, clove, green tea

## Abstract

Many current food and health trends demand the use of more ecological, sustainable, and environmentally friendly techniques for the extraction of bioactive compounds, including antioxidants. However, extraction yields and final antioxidant activities vary between sources and are highly influenced by the given extraction method and nature and ratio of the employed solvent, especially for total polyphenols, flavonoids, and anthocyanins, which are well recognized as natural antioxidants with food applications. This review focused on the most common extraction techniques and potential antioxidant activity in the food industry for various natural antioxidant sources, such as green tea, rosemary, clove, and oregano. Green extraction techniques have been proven to be far more efficient, environmentally friendly, and economical. In general, these techniques include the use of microwaves, ultrasound, high hydrostatic pressure, pulsed electric fields, enzymes, and deep eutectic solvents, among others. These extraction methods are described here, including their advantages, disadvantages, and applications.

## 1. Introduction

The main mechanisms associated with food spoilage include microbial growth and chemical oxidation. Oxidative deterioration causes the loss of aroma, taste, color, appearance, and nutritive quality because of complex reactions that produce free radicals and reactive oxygen species that alter lipids, proteins, pigments, and carbohydrates. The strategy to prevent or delay oxidation reactions involves the development of thermal or non-thermal preservation processes, in combination with an oxygen exclusion system and the use of antioxidants. The term ‘food antioxidant’ is generally applied to substances that inhibit oxidation reactions regardless of the mechanism of action. Consequently, researchers looking for strategies to inhibit oxidative deterioration are primarily focused on the incorporation of antioxidants that can disrupt the free radical-mediated oxidation chain, scavenging species that initiate oxidation, or inhibiting the generation of reactive species derived from oxygen (ROS) or nitrogen (RNS), among other mechanisms, thus prolonging the shelf life of food products.

Synthetic antioxidants, including butylated hydroxyanisole (BHA) and butylated hydroxytoluene (BHT), among others, are commonly used to inhibit oxidation in food processing, but their safety in the food industry has been questioned. In contrast, the use of ‘natural’ ingredients and additives with common names for clean labeling is perceived as healthy, and such practice in combination with the use of environmentally friendly processes is widely preferred by consumers. In addition, natural antioxidants offer several health benefits such as neutralization of the adverse effects that oxidative stress causes in cells and, thus, maintaining a stable redox state. As a result, natural antioxidant consumption has become a trend and the incorporation of natural antioxidants has increased in foods [1]. Likewise, they are also increasingly used as natural preservatives in foods.

Natural antioxidants used in foods include polyphenols, carotenoids, xanthophylls, and others extracted from natural sources, such as red fruits and grapes, and even from agro-industrial wastes such as the peels and seeds of fruits and vegetables [2]. In addition, aromatic spices such as clove, oregano, garlic, cinnamon, and green tea, among others, constitute important sources of natural antioxidants.

Conventional processes for extracting natural antioxidants such as maceration and Soxhlet distillation processes, both at the laboratory and industrial levels, involve time-consuming processes in which the yields vary according to the combination of the temperature, time, and solvent conditions, which may promote certain reactions and result in a reduction in the antioxidant capacity. Other disadvantages of conventional extraction methods involve the use of organic solvents and alkali hydroxides that may need to be eliminated via purification and fractionation. Some solvents can be toxic and represent a risk for consumers and operators, but also represent a source of pollution to the environment. As a result, high-efficiency, non-toxic techniques that involve lower solvent usage while being environmentally friendly are increasingly being studied and implemented. Among these techniques, high hydrostatic pressure, pulsed electric field, ultrasound, shock wave-assisted, enzyme-assisted, and microwave-assisted extraction methods are frequently used, as well as eutectic mixtures and supercritical fluids that have been used alone or in a combination with the aforementioned techniques. These methods have shown better extraction efficiencies than the conventional ones, with less time required and higher antioxidant activities, and, in some cases, the selective extraction of compounds [3,4,5,6,7]. These methods are more ecological and economical in the long term. However, the scaling of processes at an industrial level may not be easily achieved [8]. Furthermore, the pulsed electric field and enzyme-assisted extraction techniques can be used as preparatory or adjuvant techniques in an antioxidant extraction process and they can be combined with conventional and non-conventional techniques of extraction. Moreover, the use of these extraction techniques, in addition to being ecological and efficient, allows the extraction of active molecules with lower structural modification and great efficiency for use in various health treatments associated with oxidative stress [9,10].

In this review, the advances of environmentally friendly or green techniques for the extraction of antioxidants (focusing on the last five years, although not exclusively) are summarized, including their advantages and disadvantages (Table 1), as well as their applications in the extraction of antioxidants from spices and foods, such as clove, oregano, green tea, and rosemary, also with comparison between them.

## 2. Environmentally Friendly Techniques in the Extraction of Polyphenols

### 2.1. Microwave-Assisted Extraction (MAE)

Microwave-assisted extraction techniques use magnetic and electric fields that oscillate between 0.3 to 300 GHz, which are considered non-ionizing waves that circulate perpendicularly between them. The distribution of waves in the material or on the surface depends on the given dielectric properties, molecule polarity, and interfaces between materials and the spaces in which they are located. When waves interact with polar compounds, they can generate heat that is transmitted ionically or via dipole rotation. Heat induces breakdown in hydrogen bonds and ion migration, thus increasing the solvent penetration within the sample. This action facilitates extraction and reduces the time and amount of solvent required, thus increasing the yield and saving energy [11,12].

There are several variants of MAE as this method can be combined with other techniques such as ultrasound, vacuum, high-pressure, and reflux techniques in combination with nitrogen or other atmospheres. The incorporation of cooling systems is also common to avoid thermal degradation during the extraction and maximize the extraction efficiency [11]. However, the extraction parameters such as the microwave power, time, polarity of the solvent, particle size, and the solute–solvent ratio need to be optimized as they are the main factors involved in the MAE extraction efficiency of phenolic compounds. Solvents with high dielectric constant rapidly absorb the microwave energy such as water, ethanol, and methanol, among others. In general, hot water is the most common solvent for extraction of phenolic compounds, but as the intrinsic solubility varies with time and temperature the use of aqueous ethanol up to 70% increases the extraction of phenolic compounds. Higher levels of ethanol may induce polyphenol degradation due to the combined effect of temperature and pressure increase, as ethanol absorbs energy faster than the raw material and induces degradation and oxidation reactions, as phenolic compounds are relatively stable at 50–60 °C for short-time extraction periods, with 5 min being the extraction time recommended for extraction of phenolic compounds with the maximum antioxidant capacity. That may be related to an extraction equilibrium between the matrix and the solvent, explained by the Fick’s second law of diffusion. Larger MAE exposure induces enzymic degradation and oxidation that reduces the phenolic yield extraction [13,14].

There are authors who claim that MAE techniques for short time periods are some of the best techniques for the extraction of compounds with antioxidant capacity, such as rosmarinic acid from *Lamiaceae* plants such as rosemary, oregano, peppermint, and thyme [15].

### 2.2. Ultrasound-Assisted Extraction (UAE)

Ultrasound-assisted extraction is a technique based on the phenomenon of cavitation or the mechanical waves produced by high-frequency pulses. High-intensity ultrasound waves are suitable for extraction procedures and operate at frequencies in the range of 20–100 kHz with intensities from 10–1000 W/cm^2^ [16]. The high-cavitation energy produces shear forces in solid/liquid media and leads to an increase in the mass transfer by developing microchannels, sample erosion, and fragmentation, along with the generation of macroturbulence and mixing. All of these effects improve the solvent contact and penetration [17]. The frequency range used in UAE techniques is 20 to 40 kHz. The compression and rarefaction cycles produced by the propagation of the waves in various media induce the displacement of the molecules from their original position via cavitation bubbles, which at high intensities can coalesce and increase the temperature and local pressure, thus inducing biochemical reactions in surrounding areas and even cell fragmentation in certain tissues [18].

It is important to mention that the UAE technique is efficient as long as the parameters and conditions in which a test is carried out are optimal and that they depend on the type of sample in which the antioxidant compounds are to be extracted. It has been reported that this technique is less efficient in the extraction of polyphenols compared to a conventional technique (21.2 mg gallic acid equivalents (GAE)/g dry matter (dm) with conventional extraction vs. 15.8 mg GAE/g DM with UAE, using water as solvent, in leaves of fig tree), due to the intensity of the cavitations and, therefore, to the increase in the turbulence of the medium, which in some matrices is favorable when extracting compounds that are closely linked to their matrix and in other cases the increase in temperature and the compression phenomenon and expansion does not allow efficiency in extraction. Even the type of antioxidants that are extracted by this technique differ in type and concentration and, therefore, their antioxidant or other antimicrobial effect also differs significantly [19].

As will be seen later, ultrasound has been combined with various techniques to increase extraction efficiency, such as with supercritical fluids or even as a pretreatment technique for samples that are to be extracted with conventional techniques [20].

### 2.3. Pulsed Electric Field Extraction (PEF) and High-Voltage Electrical Discharges (HVED)

Pulsed electric field (PEF) extraction is a non-thermal process that uses high-voltage pulses in the range of 20–80 kV/cm and induces destabilization of the cell membrane by increasing its permeability, thus facilitating the extraction of intracellular components with greater efficiency. The application of an electric field on both sides of the cytoplasmic membrane acts as a capacitor inducing a transmembrane voltage variation. When a certain threshold is reached, electroporation occurs by changing the permeability with the consequent exchange of ions, macromolecules, and other cell components without selectivity [21].

High-voltage electrical discharge techniques involve electrical breakdown in liquids, which has various side effects (both physical and chemical), among which shock waves, turbulence, UV radiation, bubble cavitation, and the formation of free radical species stand out. The relevance of the technique is to find adequate conditions for the process to only induce cell rupture and not induce oxidation of the compounds, as is the case of polyphenols or other antioxidants [22].

Both techniques are efficient in the extraction of antioxidants, since in certain studies, such as in the extraction of polyphenols from blueberries, almond red leaves, lycopene, and lemon residues, among others, the yield increases by almost 300% more than conventional techniques [22,23,24,25].

An advantage of these techniques is pretreatment prior to extraction or as a replacement for the thermal drying process, thus increasing the extraction efficiency of polyphenols or other compounds. It has been reported that a drying pretreatment of fresh tea leaves using the PEF technique (1.00 kV/cm electric field strength, 100 pulses with a 100-μs pulse duration, and 5 s pulse repetition) increases the extraction rate by around two-fold, also reducing the time and the energy used by the drying technique [26].

Even for other polyphenolic extracts and flavonoids, such as those of onions, the content increases between 2.2 and 2.7 times, respectively, with respect to treatment without pulsed electric fields [27].

### 2.4. Enzyme-Assisted Extraction (EAE)

The enzyme-assisted extraction (EAE) technique is used as a pretreatment in the extraction of various compounds since it allows rupturing of the cell wall for plants, fungi, or microorganisms and becomes more efficient when using a combination of two or more enzymes. It has been reported for EAE methods that the viscosity and destabilization of emulsions can be reduced, which allows easier extraction of essential oils [28] and, therefore, antioxidant compounds.

This technique has been used to extract non-extractable polyphenols and even higher contents of proanthocyanidins compared to other conventional techniques [29]. It is even capable of being combined with the microwave technique to produce extracts with a higher concentration of polyphenols and increase the extraction of certain compounds with antioxidant relevance, such as phenolic acids and phenolic alcohols, additionally reducing the exposure time in the microwave process and featuring less solvent use [30,31].

In general, the EAE technique improves the extraction efficiency of polyphenols and antioxidant compounds of the MAE technique with a yield extraction that is 1.75 times greater than that when using other solvents. However, extraction temperature plays an important factor since the enzymatic activity may decrease at low or high temperature and efficiency will fluctuate according to the enzyme type, specificity, and concentration [28].

### 2.5. High Hydrostatic Pressure Extration (HHPE)

Extraction by high hydrostatic pressure is a technique that involves the introduction of a sample in a chamber with a high pressure, usually 100 to 1000 MPa or higher, depending on the exposure time, at temperatures from 50–200 °C for short time periods (5–10 min). The process is based on the isostatic principle and the Le Chatelier principle, where the pressure at any point in the system is the same. The chambers used vary in volume and can range from milliliters to liters (around 525 L on the industrial scale). The pressure-transmitting medium can be water, ethanol, glycerol, or silicon oil, among others [32]. During the extraction process, the desired pressure is maintained, allowing the solvent to be in a liquid state at elevated temperature near their supercritical region, which raises the solubility and diffusion properties, thus increasing the contact and penetration of the solvent within the sample [33].

The solvents used in this technique vary from aqueous ethanol, methanol, hexane, and acidic solutions, among others, However, the use of hydrophobic organic solvents may not be recommended for high-moisture samples, as water reduces the interaction of the solvent within the sample matrix [33,34].

Variations within the HHPE extraction are also known as accelerated solvent extraction (ASE), pressurized liquid extraction (PLE), high-pressure solvent extraction (HPSE), high-pressure, high temperature solvent extraction (HPHTSE), pressurized hot solvent extraction (PHSE), and subcritical solvent extraction (SSE), among others, since it involves a series of diverse techniques that combine temperatures of 50 to 200 °C, pressures of 500 to 300 psi, and application in short periods of time of 5 to 10 min. In order to consume less solvent, as well as a shorter sample preparation time, it even allows the solvent to remain in a liquid state and increases the contact of the solvent with the sample and the solubility of the compounds [34].

HHPE techniques are recognized by the Food and Drug Administration (FDA) as an environmentally friendly processes because they are processes that work at ambient or low temperatures [35]. However, the main disadvantages include the high equipment initial inversion and the possible use of larger volumes of solvents [34].

The technique can destabilize ionic bonds, hydrogen bonds, and hydrophobic bonds [36], thus inducing structural changes in the membranes and cell walls of materials from which they are going to be extracted. The structures of low-molecular-weight compounds are not altered, such as pigments, vitamins, and some polyphenols, among others. The success of the technique depends on several factors, such as the exposure time, solid and solvent radii, pH, solvent type, and solvent concentration, as well as the desired component to extract in terms of whether solubility is modified with pressure [32].

This technique increases the extraction yields of various bioactive compounds such as rutin, flavonoids, lycopene, and polyphenols, in general, with less extraction time compared to conventional techniques and with a lower solvent usage [37,38,39]. This technique has also been combined with others, such as the use of enzymes to increase polyphenol extraction efficiency, since increases in the enzymatic activity of polygalacturonase, carboxymethylcellulase, and β-glycosidase has been reported due to the action of high pressures [40].

Polyphenolic and antioxidant extracts resulting from this technique present better antioxidant activity and, when studied in in vitro analysis, they present good bioaccessibility and decrease the viability of cancer cells [10,41]. However, due to the type of equipment used, there are few studies that exist regarding the application of this technique with the spices that this review covered.

As for the types of solvents that can complement the aforementioned techniques, there are supercritical fluids and deep eutectic solvents (DESs), among others, and we will briefly describe the latter, as well as their role for antioxidant extraction.

### 2.6. Deep Eutectic Solvents (DESs)

Advances in the replacement of organic solvents have used mixtures of heterocyclic cations and organic or inorganic anions with melting points below 100 °C, known as ionic liquids (ILs), mainly derived from a charge delocalization, as the charge of both the cation, and the anion is distributed along the molecule by resonance. Ionic liquids are also known as neoteric solvents, designer solvents, ionic fluids, and molten salts. The strong ionic interaction results in a negligible vapor pressure with high-thermal stability, which constitutes a group of non-conventional solvents, further divided into task-specific ILs, room-temperature ILs and polyionic ILs [42,43,44]. Deep eutectic solvents (DESs) correspond to an alternative for ionic liquids; but some authors also consider DESs a sub-type of ionic liquids as they have similar properties [43].

DESs are obtained from the mixture and heating of two or more distinct components, Lewis or Bronsted acids, and bases to form a homogenous solution with large and asymmetric ions in a molar ratio mixture that results in a lower melting point than that of each individual component. In other words, DESs come from the combination of non-toxic, easily accessible, cheap, and sustainable hydrogen bond acceptors (HBAs) and hydrogen bond donors (HBDs). The reduction in the melting point comes mainly from the formation of hydrogen bonds and Van der Waals interactions [45]. Apart from the melting point, both DESs and ionic liquids present variations in other physicochemical properties such as viscosity, density, conductivity, acidity, surface tension, volatility, and the freezing point, which are affected by the type of co-solvent, either water or an organic solvent [43].

Common DES mixtures include quaternary ammonium salts as HBA, complexed with metal salts acting as HBD [46]. Another common HBA compound is choline chloride, while other HBDs include acetic, lactic, and oxalic acids, along with glycerol, xylitol, sorbitol, zinc chloride, butanediol, and urea. According to the composition, there are four types of DESs mixtures: (1) quaternary salt with metal chloride, (2) quaternary salt with a hydrated metal chloride, (3) quaternary salt with a hydrogen bond donor, and (4) metal chloride with a hydrogen bond donor. The first two mixtures are used to synthesize hydrophilic DESs, while mixtures 3 and 4 generate hydrophobic DESs [47].

The advantage of using a DES, with respect to other solvents, is that DESs have similar physical properties with low vapor pressure and low flammability and, in addition, they are cheaper, less dangerous, stable, biodegradable, easy to prepare, and customizable according to their application. However, the efficiency of DESs may be affected by pH variation [43].

DESs are gaining relevance in the field of the extraction of various compounds, such as proteins and the reuse of agro-industrial waste, among others. For example, the extraction of antioxidants and their health benefits have been optimized, such as blueberry extracts in the treatment of gastric ulcers without the need to remove the solvent from the extract [48].

These solvents can be combined with other techniques such as ultrasound-assisted techniques, thus increasing the polyphenol extraction performance and stabilization in comparison to other common solvents such as ethanol, increasing its half-life from 7 to 49 days for extraction with 1,2-propaneidol-choline chloride-water [49,50]. In addition, the use of DESs improves the extraction of antioxidants from other techniques such as ultrasound techniques, in which it increases the efficiency of extraction for polyphenols and the antioxidant capacity compared to the same technique with other solvents such as ethanol [51].

## 3. Application of Environmentally Friendly Techniques in Some Food Species

Table 2 shows some studies of the extraction of polyphenols and other compounds, as well as antioxidant capacities, for some spices that are used in food through the application of environmentally friendly techniques. Table 3 shows the main compounds and their chemical structures, which have been studied with environmentally friendly techniques.

### 3.1. Clove (Syzygium aromaticum)

The dried flower buds of *Syzygium aromaticum* have a distinctive flavor, and cloves have been pointed out as one of the most relevant spices due to their aromatic quality, as well as their antioxidant, antimicrobial, anti-inflammatory, and other biological properties. These properties are mainly associated with the presence of eugenol and other phenolic compounds, including flavonoids, quercetin, and kaempferol, as well as other phenolic acids (ferulic, caffeic, ellagic, and salicylic acids). However, eugenol, eugenol acetate, and β-caryophyllene are the major components of cloves [63,64]. For the extraction of essential oil from cloves and eugenol, techniques such as hydrodistillation and steam distillation are used, where volatile compounds can be lost in the process, along with high energy expenditure, the hydrolysis of compounds, and high water consumption. So, alternatives that are more sustainable and ecological have been implemented [65]. It has been reported that incorporating the microwave technique as an extraction method during hydrodistillation and steam distillation reduces the extraction time by between 4 and 4.8 times without modifying the chemical composition or antioxidant capacity of the final extract [65].

#### 3.1.1. MAE

Using the MAE technique, the extraction of clove oil gives a yield of 13.11% (*w*/*w*), with 11.93% of eugenol (*w*/*w*) when 30 g of dried clove buds are used with 200 mL of water per 30 min [66]. In this same study, they reported that the use of MAE for extraction reduces the use of water, the energy required (600 W), and the emission of CO_2._

The MAE technique and its modifications extract certain compounds with a difference in efficiency compared to common thermal extraction techniques. An example of this is in the case of eugenol, since 66.9% was obtained by means of the coaxial MAE technique for 30 min, while 87.1% was obtained via conventional distillation (for 180 min). However, the extraction of compounds such as caryophyllene increases from 6.4% to 24.8% with the MAE method, and other compounds of biological relevance that do not appear by conventional techniques are extracted [67]. Even the MAE technique extracts oxygenated monoterpenes such as cineole and β-myrcene more efficiently [68].

The clove extracts obtained by the supercritical fluid extraction technique have higher contents of eugenol and eugenol acetate, representing the highest antioxidant capacity, obtaining 86.70% for the former and 13.30 for the latter, while, for the MAE technique, 81.47% and 8.11% were obtained, respectively, although other compounds such as caryophyllene have been obtained at 1.32% [69].

#### 3.1.2. UAE

In the case of cloves, ultrasound techniques have been combined with supercritical fluid techniques, obtaining high yields in the extraction of oil from cloves, as well as in the presence of important components with antioxidant activity such as eugenol and eugenyl acetate when compared to other conventional techniques such as heat reflux, steam distillation, and even microwave-assisted hydrodistillation and microwave-assisted extraction, where the process allows a better extraction of α-humulene [70], although scaling still remains an important area of opportunity for this technique.

#### 3.1.3. DESs

There are studies where the extraction by a DES has been used to increase the performance of terpene extraction for multiple uses and from different spices such as cinnamon, cumin, fennel, clove, and thyme [71]. Being the solvent formed from tetrabutylammonium bromide and dodecanol (1:2 molar ratio), it is the solvent that extracts compounds such as eugenol (1,226,059 ± 36,216 µg/g), caryophyllene (289,518 ± 7437 µg/g), and α-humulene (38,483 ± 1588 µg/g) from cloves, among other compounds, such as α-terpineol, β-farnesene, linalool, anethole, and cuminaldehyde in lower concentrations [71].

### 3.2. Green Tea (Camellia sinensis)

Green tea is a drink obtained from the leaves of *Camellia sinensis* and widely recognized as an excellent antioxidant due to the amount (i.e., type and concentration) of polyphenols. Green tea is associated with various beneficial health effects in humans, such as the reduction of certain types of cancer and cardioprotective effects [72,73,74]. Hence, its consumption and obtaining extracts with a higher content of polyphenols, including catechins such as epigallocatechin gallate, epigallocatechin, epicathechin, and gallic acid, among others, have become especially important [75].

#### 3.2.1. MAE

A comparison between MAE and ultrasound was made regarding the extraction of total polyphenols and their antioxidant activity, where MAE was found to be more efficient with 125 ± 5 mg gallic acid/g dry weight and 56 mg/g of phenol of 2,2-diphenyl-1-picrylhydrazyl free radical assay (DPPH) inhibition 50%, while the ultrasound extract presented 96 ± 6 mg gallic acid/g dry weight of total polyphenols and 66 mg/g of phenol in the inhibition of DPPH [76].

#### 3.2.2. UAE

The use of UAE together with a clarification technique allows a better extraction of polyphenols such as epigallocatechin gallate, epicatechin, epigallocatechin, catechin, gallic acid, and caffeine, where the optimal extraction conditions were 77 °C in a tea-to-water ratio of 73 g/L with 77% amplitude, allowing the extraction of total polyphenols (12,318 ± 87 mg GAE/L) and flavonoids (3774 ± 28 mg rutin equivalent/L) [77].

Despite the advantages of environmentally friendly techniques such as UAE, they must be optimized to achieve the best quantity and quality for bioactive compounds such as antioxidants. In a study comparing UAE with water, UAE with ethanol, and the hot water technique, UAE with ethanol was highly effective in extracting some compounds such as epigallocatechin gallate (7.62 ± 0.03 g/100 g green tea), followed by UAE with water (4.81 ± 0.04 g/100 g green tea), and, finally, the conventional method with hot water (3.86 ± 0.02 g/100 g green tea). However, when scaling the process, the efficiency of the conventional process was higher than that of the UEA process (3810 ± 0.26 vs. 2460 ± 0.06 g/100 g, respectively) [78].

In addition, this technique has also been used for the extraction of polyphenols from various types of tea (black tea, mate tea, blackberry, and green tea) by combining experimental conditions such as type of solvents, such as water, ethanol, and methanol, and exposure time in the ultrasound, reporting that the optimization in the extraction is unique for each sample, but that the solvent that improves the extraction most through this technique is 50% ethanol and 50% methanol [55].

#### 3.2.3. PEF

This technique for green tea has been used as a drying and pretreatment method for the extraction of polyphenols from the leaves, in which it was shown that a greater intensity in the electric field and a larger number of pulses (1.25 kV/cm, 200 pulses) improves the efficiency in the extraction of polyphenols (by 2.75 times vs. the low intensity and lower number of pulses of 0.75 kV/cm and 10 pulses, respectively) with the cellular rupture of the leaves, although this process increases the temperature around 9.1 °C, which may not be favorable for the extract compounds [26].

#### 3.2.4. HHPEE

This technique has shown higher efficiency in the extraction of polyphenols from green tea leaves, using shorter time periods, such as for 1 min, with extraction efficiencies similar to the traditional methodologies at room temperature for 20 h, along with that of ultrasound for 90 min and heat reflux for 45 min [79].

#### 3.2.5. DESs

The extraction of polyphenols from green tea using DESs has shown good extraction efficiencies, reaching 97% for epigallocatechin gallate and up to 82.7% for catechin [80].

One of the interesting properties of drinks such as teas and that can be a quality control of the drink is the concentration of catechins. Hence, their extraction and quantification has become relevant. For this, the DES technique (malic acid and Girard’s reagent T in a 2:1 ratio at 50 °C for 50 min) has been used successfully, obtaining around 63.1 ± 1.04 µg/g of dry weight of epigallocatechin gallate up to 19.8 ± 0.06 µg/g of dry weight of gallocatechin, and other catechins in lower proportions [81]. In the same sense, the extraction of total polyphenols using choline and glycerol (1:2 molar ratio) and ultrasonication for 21 min has allowed the extraction of 243 ± 7 mg gallic acid equivalent/g dry weight with an antioxidant activity of DPPE of 215 ± 6 (mmol Trolox/100 g dry weight) against 219 ± 3 of polyphenols with 195 ± 3 of activity when using the same extraction technique with ethanol, which indicates that it extracts more polyphenols and with greater antioxidant activity [51].

Even the use of DESs (malonic acid and vinylpyrrolidone) allows coupling with other techniques such as immobilization on magnetic particles of molybdenum disulfide to increase extraction efficiency and selectively acquire compounds such as epigallocatechin gallate from green tea [82]. A similar technique has been studied for the extraction of compounds such as theobromine, caffeic acid, theophylline, and catechin hydrate through the use of ternary deep eutectic solvent magnetic molecularly imprinted polymers with specific efficiencies higher than 89% [83].

### 3.3. Oregano (Origanum vulgare L.)

The term oregano refers to a set of 60 species of 17 genera of the *Verbenaceae* and *Laminaceae* families that present a characteristic smell and taste. Greek oregano (*Origanum vulgare*) is well known for its high content of rosmarinic acid [84]. Various herbs that make up oregano have anti-inflammatory and antioxidant activities, which is why they have been used in the control, prevention, and reduction of side effects of some diseases, such as diabetes, asthma, indigestion, headaches, and stomach pain, among others [85], due to the content of flavonoids and phenolic acids, which have relevant antioxidant activity, such as naringenin, apigenin, caffeic acid, chlorogenic acid, and quercetin, among others [84].

#### MAE

In a study carried out on various plants, including oregano, a comparison was made for the extraction of rosmarinic acid (which has diverse antioxidant properties) between MAE, heat reflux, and maceration with stirring, finding that the MAE technique presented the same efficiency for extraction with the other techniques, but with the advantage that it occurs in a shorter time [15]. In addition, an important factor in this technique is temperature since high temperatures (above 50 °C) can cause changes in the stability of certain compounds, such as rosmarinic acid, and especially if a high temperature is prolonged.

### 3.4. Rosemary (Rosmarinus officinalis L.) 

Rosemary is an ornamental and aromatic herb of Mediterranean origin that is used in various regions in food. Rosemary has been reported to have important antioxidant and antimicrobial activities, which have been associated with the presence of various polyphenolic compounds such as diterpenes and phenolic acids. These compounds have also been studied for their health effects, which include anti-hyperglycemic, anti-cancer, and metabolic syndrome therapeutic effects, among others [86,87,88].

#### 3.4.1. MAE

Extraction using MAE has already begun to be used in the formulation of food products, offering good final sensory properties for products and additional health benefits if extracts are focused on the extraction of antioxidant and phenolic compounds. Such is the case of the incorporation of rosemary extract (total polyphenols of 150.16 ± 1.18 mg GAE/g dry weight (dw)) in fresh cheese, whose extraction process by MAE (78.16% ethanol, microwave power 351.82 W, extraction time 122.65 s) offers acceptable flavors and aromas to the product in addition to antioxidant properties [89].

#### 3.4.2. UAE

The UAE technique with rosemary allows greater efficiency in the extraction of carotenoids when 50% ethanol is used (about 0.12 mg carotenoids/g dw) and, therefore, a considerable antioxidant activity (approximately 7 mmol Trolox), with total polyphenols of approximately 22 mg GAE/g dw and flavonoids of 19 mg catechin equivalents/g dw [90]. Other UAE optimization data for rosemary suggest using ethanol at a concentration of 55.19% for 12.48 min at 200 W, presenting a total extraction yield of 20.82 ± 0.44 g of dry extracts per 100 g of dry weight of rosemary leaves, a total phenolic content (TPC) of 185.16 ± 4.03 mg GAE/g of extract dw, and IC50 805.84 ± 4.17 of DPPH [91]. Even this technique increases the extraction of carnosic acid by 13% and rosmarinic acid by 6.8% when using n-hexane and 19.5 kHz (140 W) [92]. At this point, there is controversy since other studies mention that the ultrasound technique improves the extraction of carnosic acid, while the microwave technique is better for rosmarinic acid [93].

The interesting thing about this technique is that, when combined with traditional methods with heat reflux, it increases the yields in the extraction of phenols by up to 103.44 ± 2.12% when ultrasound is applied for 15 min with 30% of the maximal ultrasonic power [20].

Even this technique allows the extraction of polyphenolic compounds from rosemary, even when the raw material has already been used for the extraction of essential oils, obtaining polyphenols with 77.5 ± 1.2 mg GAE/g dw with an antioxidant capacity of 37.8 ± 1.1 mg GAE/g dw and 47.4 ± 1.1 (mg/g dw) of total phenolic diterpenes for UAE with 60% ethanol with a frequency of 37 kHz at 22 °C [57].

The application of rosemary extracts using the ultrasound technique, when incorporated into hamburger meat, reduces the sensory descriptors related to lipid oxidation as well as the presence of volatile compounds resulting from the oxidation process [94].

#### 3.4.3. EAE

This technique has been implemented for the specific extraction of rosmarinic acid, since the use of enzymes, specifically cellulase A, for 4.63 h at a concentration of 2.56% at 36.6 °C increases the extraction efficiency (by 1.61 times vs. the control without enzyme) with an IC50 antioxidant capacity of 532.01 μg/mL with the DPPH technique. In addition, the use of other enzymes (bromelain, papain, and Champzyme FP) has been explored for the extraction of this same compound, although with lower efficiencies compared to cellulase (differences between 1.32 and 1.17) [95].

#### 3.4.4. DESs

The extraction of total polyphenols from rosemary leaves using choline chloride and 1,2-propaneidol at 65 °C in a liquid-to-solid ratio of 40:1 resulted in a 78-mg gallic acid equivalent with an antioxidant activity of 80-mg equivalents of Trolox [96], where the proportion of total polyphenols extracted was approximately 220% higher compared to the control in which 70% ethanol was used.

In this sense, the extraction of polyphenols with DESs (choline chloride and glycerol and lactic acid and choline chloride) in combination with ultrasound-assisted techniques presents the highest antioxidant capacity activity as determined by the DPPH technique compared to ethanol or other DESs’ solvents (increasing from 132.19 to 155.83 mM Trolox equivalent/g plant material) [50].

A DES variant is one that involves a eutectic mixture of only natural compounds such as amino acids, sugars, and organic acids, among others, which makes them more ecological biodegradable and even economical. These mixtures are called natural DESs or NADESs. NADESs have been used in the extraction of flavonoids from rosemary, and specifically the combination of acetylcholine chloride and lactic acid (ratio 2:1) has allowed the extraction of rutin, naringin, hesperidin, neohesperidin, naringenin, and hesperidin (11.0, 12.9, 14.4, 11.4, 16.9, and 156 µg/g dw, respectively) in concentrations similar to those of other conventional heat techniques, but with less time required and with a more environmentally friendly technique [97].

## 4. Conclusions

Several alternative environmentally friendly techniques’ extraction technologies offer new opportunities for process development to improve efficiency and yields for the extraction of several biologically active compounds, such as natural antioxidants, within application in foods. These technologies offer several advantages, such as better extraction efficiencies than the conventional technologies, along with selective and higher antioxidant activities, although some technological and commercial constraints need to be resolved, particularly regarding process automation and control systems. The main advantages include lower energy consumption, lower costs, and the use of less toxic and sustainable solvents, which in combination lead to the reduction of greenhouse gas emissions and a smaller carbon footprint.

Therefore, the environmentally friendly techniques constitute a promising technology for extraction of several compounds including those antioxidants from natural sources such from green tea, rosemary, clove, and oregano. These natural products are rich in phenolic compounds and it is necessary to ensure their effective extraction with minimal degradation as they are thermally unstable. Furthermore, these environmentally friendly processes enhance the solvent diffusion through the vegetable materials and may be used in combination to improve the extraction yield in comparison with conventional extraction methods. Furthermore, the environmental techniques may be applied for extraction of several bioactive compounds including those phenolic compounds from other underutilized sources, allowing the reutilization of agro-industrial byproducts.

However, further research needs to be undertaken to establish the specific operation conditions, as well as to establish the effect of time and temperature or other factors in the extraction yield, in addition to the impact of particle size, pH, or moisture of the raw materials, for a better understanding of the solute-solvent interaction. For instance, the efficiency of DESs is highly affected by variation in the pH as changes in the ionic species are less predictable in non-aqueous systems. Although several authors claim a synergistic action by the combined use of some environmentally friendly techniques and other emerging procedures, the extraction efficiency needs to be verified for each material and process; for instance, the combined action of EAE and MAE techniques must be carefully designed to avoid enzyme deactivation.

Finally, environmentally friendly techniques may be employed in combination with other strategies, such as micro- and nanoencapsulation, for the stabilization of the final product that preserves the antioxidant properties, thus facilitating the incorporation of the natural antioxidants into the food industry in favor to the consumer’s health.

## Figures and Tables

**Table 1:** Advantages and disadvantages of assorted environmentally friendly techniques for antioxidant extraction.

Method	Advantage	Disadvantage
Microwave-assisted extraction (MAE)	Short time (15–30 min). May or may not use a solvent other than water Low solvent usage Easy industrial escalation Low power consumption Low levels of CO_2_ released into the atmosphere Non-contact heat source Accelerates mass and energy transfer	Needs a solvent separation method. It can affect thermolabile metabolites and in some cases causes oxidation Non-selective extraction
Ultrasound-assisted extraction (UAE)	Uses room temperatures. Less energy Lower solvent volume	Difficulty scaling Decrease in its efficiency in systems with high viscosity Temperature stability Solvent contamination Non-selective extraction
Pulsed electric field extraction (PEF) and high voltage electrical discharges (HVED)	Low energy Continuous operability Short times	Difficulty scaling Possible oxidation of compounds (HVED)
Enzyme-assisted extraction (EAE)	Easy operation and high specificity if the choice of enzymes is right High efficiency Environmentally friendly Low energy requirements and low operating temperature	Scaling and influencing factors such as enzyme concentration, oxygen, pH, temperature, and agitation Establishment of operating conditions if two or more enzymes are used in the process
High-hydrostatic pressure extraction (HHPE)	Can be operated at room temperature or in refrigeration temperatures Short operation time Less solvent use compared to heat techniques Better quality, efficiency, and biological activity for extracts Reduced extraction times	May promote oxidation reactions At an industrial level it is a semi-continuous or batch process
Deep eutectic solvents (DESs)	Biodegradable solutions Non-toxic Easy to prepare	Expensive to scale The final solution possesses high viscosities and densities

**Table 2:** Polyphenol extraction from some spices using environmentally friendly techniques.

Material	Method	Experimental Conditions	Total Phenolic Content	Other Relevant Data	Reference
Clove	UEA (batch reactor)	1 kg in 20 L ethanol/water (1:1) in 45 min at 25 kHz effective power 360 W at 28–30 °C	Around 195 ± 1 mg GAE/L extract	Around 45% in inhibition of DDPH	[52]
	UEA (multi-horn flow reactor)	1 kg in 20 L ethanol/water (1:1) in 45 min at effective power 350 W, four horns of 21.0 kHz, flow 1350 mL/min	215 ± 3 mg GAE/L of extract	Around 52% in inhibition of DDPH	[52]
	Conventional method	95% ethanol at room temperature with agitation for 24 h	54.3 ± 7.3 mg GAE/g	Total flavonoids: 6.9 ± 0.36 mg catechin equivalents/g DPPH (IC50) 0.45 ± 0.08 FRAP: 1216 ± 45.3 mg Trolox/g extract	Unpublished data
Green tea	MAE	350.65 W and 5 min irradiation	116.58 mg GAE/g	Total flavonoid content: 49.33 mg catechin equivalent/g, condensed tannins content: 9.89 mg catechin equivalents/g, DPPH IC50: 294.46 µg/mL	[53]
	Microwave hydro-diffusion and gravity + UEA	Leave wastes in water (15:1). Extraction conditions: 300 W irradiation + UAE at 30 min at 80 °C and 80 kHz	130 mg GAE/g extract	Antioxidant activity of 0.4 g Trolox equivalents/g	[54]
	UEA	50% methanol in an ultrasonic bath at 28 kHz for 15 min at 55°C	90.87 ± 1.52 mg GAE/g dw	Total flavonoids content: 26.18 ± 0.86 mg CAT/g dw Total antioxidant capacity: 94.18 ± 0.49% inhibition DPPH	[55]
	UEA + DES	DES solvent: choline chloride + glycerol. Ratio of liquid:solid of 36:1 (mL/g), ultrasonic power of 461.5 W with 21 min	243 ± 7 mg GAE/g dw	Important presence of 4 catechins: epicatechin, epigallocatechin, epicatechin gallate and epigallocatechin gallate. DPPH: 215 ± 6 mmol Trolox/100 g dw FRAP: 332 ± 9 mmol Fe (II)/100 g dw	[51]
	Conventional method	95% ethanol at room temperature with agitation for 24 h	108.4 ± 6.9 mg GAE/g	Total flavonoids: 32.10 ± 1.91 mg catechin equivalents/g DPPH (IC50) 0.65 ± 0.05 FRAP: 382.3 ± 58.3 mg Trolox/g extract	Unpublished data
Oregano	MAE	5 g with 100 mL of absolute ethanol for 20 min at 150 W microwave power at 60 °C	65.40 ± 1.58 mg GAE/g dw	----	[56]
	UAE	5 g with 500 mL ethanol in an ultrasonic bath for 20 min and then extraction with water at 60 °C for 1 h	56.22 ± 2.11 mg GAE/g dw	-----	[56]
	SE (Soxhlet extraction)	5 g with 200 mL of absolute ethanol for 8 h	50.88 ± 1.32 mg GAE/g dw	-----	[56]
	Conventional method	95% ethanol at room temperature with agitation for 24 h	22.8 ± 0.63 mg GAE/g	Total flavonoids: 1.60 ± 0.25 mg catechin equivalents/g DPPH (IC50) 1.08 ± 0.07 FRAP: 178.1 ± 14.8 mg Trolox/g extract	Unpublished data
Rosemary	Maceration + PEF	Wet and ground plant material exposed to 1000 pulses of 15 µsec in a field strength of 5.2 kV/cm. Extraction with 60% ethanol	64.0 ± 0.3 mg GAE/g dw	------	[57]
	MAE + microwave hydro-diffusion and gravity (pretreatment MHG)	Pretreatment MHG for 579 s (100 g fresh plant with 50% ethanol in residual water of other MHG). MAE AT 60 °C	55.5 mg GAE/ dw	------	[58]
	UAE	Extraction with 60% ethanol for 70 min with an ultrasonic bath with a frequency of 37 kHz at 22 °C	77.5 ± 1.2 mg GAE/g dw	Flavonoids: 16.1 ± 0.3 mg QUE/g dw, carnosic acid: 29.1 ± 0.9 mg/g dw; carnosol: 16.1 ± 0.7 mg/g dw; Rosmarinic acid: 10.1 ± 0.6 mg/g dw	[57]
	UAE + DES	150 mg rosemary leaves + 2.85 mL DES (choline chloride: 1,2-propaneidol) for 120 min with an ultrasound frequency of 50–60 Hz	62.21 ± 3.85 mg GAE/g plant	150.63 ± 0.3 mM Trolox equivalent/g plant for DPPH antioxidant activity and 148.24 ± 8.75 mM Trolox equivalent/g plant for FRAP activity	[50]
	Conventional method	50% ethanol at 60 °C	35.29 mg GAE/g dw	-------	[58]
	Conventional method	95% ethanol at room temperature with agitation for 24 h	14.59 ± 1.31 mg GAE/g	Total flavonoids: 2.9 ± 0.31 mg catechin equivalents/g DPPH (IC50) 4.7 ± 0.37 FRAP: 51.36 ± 1.2 mg Trolox/g extract	Unpublished data

Abbreviations: GAE: gallic acid equivalents; dw: dry weight; DPPH: 2,2-diphenyl-1-picrylhydrazyl free radical assay; FRAP: Ferric antioxidant power assay; QUE: quercetin equivalents; UAE: Ultrasound assisted extraction; MAE: Microwave-assisted extraction; DES: deep eutectic solvent; PEF: pulse electric field extraction.

**Table 3:** Main compounds and structures present in the extracts obtained by environmentally friendly techniques of some spices [59,60,61,62].

Material	Name and Empirical Formula	Structure
Rosemary	Rosmarinic acid (C_18_H_16_O_8_)	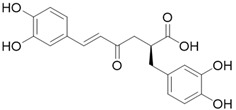
	Carnosol C_20_H_26_O_4_	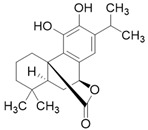
	Carnosic acid C_20_H_28_O_4_	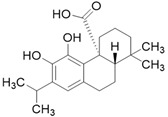
Clove	Eugenol C_10_H_12_O_2_	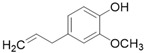
	β-caryophyllene C_15_H_24_	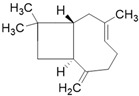
	Eugenyl acetate C_12_H_14_O_3_	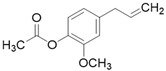
	α-humulene C_15_H_24_	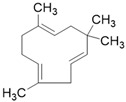
Green tea	Catechin C_15_H_14_O_6_	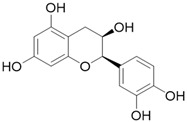
	Epicatechin C_15_H_14_O_6_	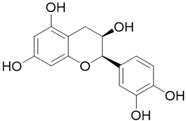
	Epicatechin gallate C_22_H_18_O_10_	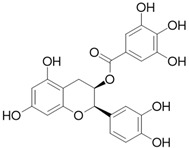
	Epigallocatechin C_15_H_14_O_7_	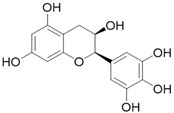
	Epigallocatechin gallate C_22_H_18_O_11_	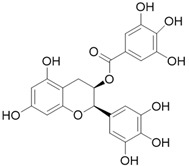
	Quercetin C_15_H_10_O_7_	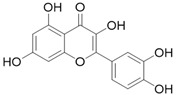
Oregano	Carvacrol C_10_H_14_O	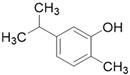
	Thymol C_10_H_14_O	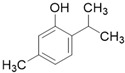
	*p*-cymene C_10_H_14_	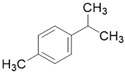
	β-caryophyllene C_15_H_24_	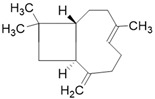

## Data Availability

Not applicable.
